# ASSOCIATIONS BETWEEN SMOKING CONSUMPTION, TOBACCO CESSATION ATTITUDES, AND AGE AMONG LATINO HEALTHCARE PROVIDERS

**DOI:** 10.1093/geroni/igz038.3270

**Published:** 2019-11-08

**Authors:** Belén Hervera, Lisette Irarrázabal, Lilian Ferrer, Rosina Cianelli

**Affiliations:** 1 University of Miami, Miami, Florida, United States; 2 Pontificia Universidad Católica de Chile, Santiago, Chile

## Abstract

Hospitalization is a good opportunity to offer smoking cessation programs to smokers. Healthcare providers′ (HCP) tobacco consumption and cessation attitudes are known to affect the provision of cessation interventions. Lesser known are Latino HCP’s tobacco intervention attitudes. This study aimed to examine the associations between tobacco cessation attitudes (TCA), levels of consumption, and demographics among Latino HCP’s. A quantitative, correlational, cross-sectional design was used. 66 HCP’s working in a public hospital in Santiago, Chile self-reported demographics (age, gender, profession), tobacco consumption, and TCA. TCA’s include questions regarding Acceptability of Brief Counseling (ABC), belief whether smoking is harmful for patients, and duty to aid patients quit smoking. Majority of HCP’s (34 years old, 83% female, 58.5% technical nurses, 38.5% nurses, 3.1% Kinesiologists) did not consume tobacco (67%). Pearson’s correlation revealed that greater HCP age was significantly associated with less belief that smoking is harmful for their patients (r = -.36, p. = .004). ABC (M = 22, SD = 5.5) was positively associated with the belief that smoking is harmful for patients (r = .306, p = .016) and duty to help patients quit smoking (r = .574, p = .000). Findings provide evidence that HCP’s TCA’s are important factors to consider during implementation of a brief counseling for tobacco cessation. Further research should focus on increasing HCP’s acceptability of providing cessation care to their patients. Specifically, tailoring education and interventions by age might serve useful to address the differences in TCA’s which may subsequently influence their tobacco cessation practices.

